# Antibody response against *Trichinella spiralis *in experimentally infected rats is dose dependent

**DOI:** 10.1186/1297-9716-42-113

**Published:** 2011-11-30

**Authors:** Frits FJ Franssen, Manoj Fonville, Katsuhisa Takumi, Isabelle Vallée, Aurélie Grasset, Marie A Koedam, Piet W Wester, Pascal Boireau, Joke WB van der Giessen

**Affiliations:** 1 Laboratory for Zoonoses and Environmental Microbiology, National Institute for Public Health and the Environment (RIVM), Bilthoven, The Netherlands; 2 Anses, ENVA, UPEC, Laboratory for Animal Health, Joint Research Unit, Molecular Biology, Parasitic and Fungal Immunology (JRU BIPAR), Maisons-Alfort, France

## Abstract

Domestic pigs are the main representatives of the domestic cycle of *Trichinella spiralis *that play a role in transmission to humans. In Europe, backyard pigs of small household farms are the most important risks for humans to obtain trichinellosis. Rats might play a role in the transmission of *Trichinella spiralis *from domestic to sylvatic animals and vice versa. In order to be able to investigate the role of wild rats in the epidemiology of *T. spiralis *in The Netherlands, we studied the dynamics of antibody response after *T. spiralis *infections in experimental rats, using infection doses ranging from very low (10 muscle larvae, ML, per rat) to very high (16 000 ML per rat). To evaluate the feasibility of rats surviving high infection doses with *T. spiralis*, clinical and pathological parameters were quantified. Serological tools for detecting *T. spiralis *in rats were developed to quantitatively study the correlation between parasite load and immunological response. The results show that an infection dose-dependent antibody response was developed in rats after infection with as low as 10 ML up to a level of 10 000 ML. A positive correlation was found between the number of recovered ML and serum antibody levels, although specific measured antibody levels correspond to a wide range of LPG values. Serum antibodies of rats that were infected even with 10 or 25 ML could readily be detected by use of the *T. spiralis *western blot 2 weeks post infection. We conclude that based on these low infection doses, serologic tests are a useful tool to survey *T. spiralis *in wild rats.

## Introduction

*Trichinella spiralis *is the only known *Trichinella *species out of 12 recognized species or genotypes [1] that is transmitted and maintained in both a domestic and sylvatic cycle. The *T. spiralis *sylvatic cycle involves omnivores like the wild boar, carnivores like the wolf and fox, but also scavenger wild rodents [2,3]. *T. spiralis *is distributed worldwide and maintained in pigs as one of the most important representatives of the domestic cycle. In Europe, free ranging pigs of small household farms are the most important risk for public health [3].

Rats play a role in the transmission of *T. spiralis *from domestic to sylvatic animals and vice versa. It has been shown that pigs exposed to rats were infected more often, whereas pigs that were physically separated from rats remained free of *Trichinella *[4]. Rats in the vicinity of pig farms were infected only when *T. spiralis *occurred in pigs on those farms under low sanitation levels [5,6]. However, it has been shown that even in the absence of a known source of infection on farm level, *T. spiralis *is able to persist in rats [5].

In the geographical spread and maintenance of *T. spiralis *in nature, humans play a major role. Disposal of infected carcasses of pigs or hunted wild boars, wolves and foxes in nature or on waste disposal sites might be a driving force in spreading *T. spiralis *infections in wild rat populations [7,8]. Circumstantial evidence has indicated that an outbreak of *T. spiralis *in outdoor farmed wild boar could be attributed to an invasion of rats from an improperly closed down landfill in the vicinity of the farm [9]. Jovic et al. [10] showed by bioassay using rats, that *T. spiralis *larvae in artificially infected pork meat that had been buried in the ground at a depth of 30-100 cm, remains infective for rats for more than 91 days.

Rats were shown to be a potential reservoir host species of *Trichinella *using mathematical models, provided that cannibalism occurs [11]. It was argued in that study that rats should be included in the minimal set of wildlife species that maintain the cycle of *T. spiralis*. Even if rats do not represent an important route of *Trichinella *distribution, but are merely sentinel species, it might be useful to monitor rats for *Trichinella *in a wildlife monitoring programme. Wildlife monitoring is one of the tools indicated by the EU regulation 2075/2005EU to control Trichinella [12]. The results of a rodent monitoring might give additional information about Trichinella dynamics in wildlife and might also be useful in a more generic wildlife monitoring programme.

In this study, we developed serological tools to quantitatively study the correlation between parasite load and immunological response of artificially *T. spiralis *infected rats at different infection levels. To augment the dynamics of *T. spiralis *in infected rats using different infection doses, and to evaluate the probability of rats surviving high infection doses with *T.spiralis*, clinical and pathological parameters are quantitatively described as well.

## Materials and methods

### Experimental infection

Male Wistar Unilever rats weighing 230-280 g were infected with *T. spiralis *muscle larvae (strain ISS 14), which had been isolated by pepsin-HCl digestion from previously infected mice or rats. To assess low dose infection, thirty-six rats were divided into six groups of six animals per group and individual rats were infected with 10, 25, 50, 100, 200 and 400 ML per group. To assess high dose infection, an additional thirty rats were divided into ten groups of three animals per group and individual rats were infected with 200, 400, 2 000, 4 000, 6 000, 8 000, 10 000, 12 000, 14 000 and 16 000 ML per group. On the first day of the experiments, all rats received ML which were delivered directly into the stomach through a gastric tube. Doses of 10 to 400 ML were prepared by counting individual larvae. Doses between 2 000 and 16 000 ML were prepared by a series of dilutions. At each dilution step, the suspensions were mixed continuously by magnetic stirring to avoid possible sedimentation of the muscle larvae. Blood from rats of both the low and high dose experiments was collected weekly via orbital puncture and sera were stored at -20°C until further use. Rats in the groups of 10 000 ML or higher showed signs of severe clinical illness and their diets were changed to soft food to prevent premature termination. Forty-two days post infection (dpi) the rats were euthanized. Body weights and clinical scores were determined daily. Clinical scores were defined as follows: 0-no signs, 1-rough coat and light diarrhea, 2-rough coat, animal inactive and severe diarrhea, 3-rough coat, inactivity, severe diarrhea and weight loss, 4-rough coat, inactivity, severe diarrhea, more than 20% weight loss and decrease in body temperature, 5-death. The experimental protocol was approved by the RIVM Animal Experiments Committee.

### Pathology

At terminal sacrifice at 42 dpi, samples of front leg muscle (1 animal per group, highest level of recovered muscle larvae), duodenum, heart, liver, thymus, spleen and mandibular lymph node were collected and fixed in 4% buffered formaldehyde. Tissues were processed to paraffin blocks and 4 μm sections were cut and stained with H&E. All organs were examined in the lowest and highest infection dose groups, and affected organs (spleen, thymus, lymph node and muscle) also from intermediate groups.

At day 8 post infection (pi), an autopsy was performed on the animals of the highest dose group (16 000 ML), of which the organs were examined as described above. In addition, lungs and three muscles (diaphragm, masseter and quadriceps femoris muscles) and the intestines were examined histologically (small intestine-4 locations, large intestine-3 locations). Selected organs were stained with PAS additional to H&E.

### Isolation of muscle larvae

From each rat, the muscle tissue was separated and *T. spiralis *ML were isolated by artificial digestion according to EU regulation 2075/2005 [12]. Briefly, after weighing the carcass, all muscle tissue from each rat was isolated, its weight was established and it was digested using pepsin-HCl for 30 min at 45 ± 1°C. After two successive sedimentation steps, the resulting suspension containing ML was transferred to Petri dishes and the number of isolated larvae was determined by two technicians. These counts were used to calculate average values and the number of larvae per gram (LPG) muscle tissue per rat.

### Production of Trichinella ES antigen

*T. spiralis *excretory/secretory (E/S) antigen was produced as described previously by Gamble [13]. Briefly, Trichinella ML (ISS 14) were isolated from mouse muscle tissue by artificial digestion and washed three times in PBS containing penicillin (12 mg per 100 mL) and streptomycin (20 mg). Washed ML were incubated at a concentration of 10 000 larvae per mL in a CO_2 _incubator for 18 h at 37°C in 250 cm^2 ^culture flasks containing 70 mL RPMI 1640 culture medium (supplemented with 1% glutamine and 1% penicillin/streptomycin, Gibco 10378, Invitrogen, Bleiswijk, The Netherlands). The culture medium was separated from the ML by centrifugation and the supernatant was concentrated in a dialysis membrane (Spectra/Por molecularporous membrane MWCO 6-8 000, Spectrum Laboratories, Inc., Rancho Dominguez, USA) by dehydration using polyethylene glycol 20 000 (PEG, Fluka 81300, Sigma-Aldrich Chemie GMBH, Steinheim, Germany). The concentrated culture medium was dialyzed two times over-night against PBS at 4°C and the dialyzed and concentrated solution containing *T. spiralis *ES proteins was further concentrated using 5 kD molecular weight cut-off filters (Amicon Ultra Centrifugal Filter Devices, Millipore, Carrigtwohill, Ireland). The protein concentration was determined by BCA protein assay (Pierce, Rockford, IL, USA).

### In-house ELISA

Antibody responses of the infected rats were determined by an in-house ELISA. Flat-bottom ELISA plates (Nunc 66904) were coated with 0.125 μg of *T. spiralis *ES antigen per well for one hour at 37°C. After washing (PBS/0.05% Tween 20 (PBS-T)) and saturation (1% BSA in PBS-T at 4°C overnight), sera were applied (100 μL per well, 1/100 dilution in PBS-T) for one hour at 37°C. After washing, each well was incubated for one hour at 37°C with 100 μL Goat anti-Rat IgG conjugated to Horse Radish Peroxidase (1/8000 in 1% BSA/PBS-T). After washing, antigen-antibody interaction was visualized by adding 100 μL Sure Blue™ TMB substrate solution (KPL, Gaithersburg, USA) per well and incubation for ten minutes at room temperature. The reaction was stopped with 100 μL 2 M H_2_SO_4 _and optical density (OD) values were determined with an ELISA reader (Bio-Tek EL808 Ultra Microplate Reader, Bio-Tek Instruments, Inc.) at 450 nm.

### Calculation of normalized OD and cut-off

Sera were tested in duplicate and normalized OD minus blank (OD_n_) values were calculated using the formula OD_n _= (OD-*b*)/*a*, where OD is the average OD minus blank value of the duplicates, *a *is the x-variable and *b *the intercept from the linear regression analysis tool in Microsoft Excel. Positive and negative rat control sera were used to determine variables *a *and *b *for each ELISA plate to correct for inter-plate and day-to-day variation. The OD_n _values of pre-immune (day 0) sera of all experimental rats was used to define a cut-off level as average OD_n _plus 2 times standard deviation.

### Western blot

*T.spiralis *ES antigen (1 μg total protein per lane) was separated by SDS-Page and transferred to nitrocellulose membrane (Trans-Blot^® ^Transfer Medium, Bio-Rad Laboratories, Hercules, CA, USA). After saturation of the membrane with 1% BSA in PBS-T (BSA/PBS-T), serum dilutions (1:50 or 1:300 (positive control) in BSA/PBS-T) were applied and immunoreactions were visualized with Goat anti Rat IgG conjugated to Horse Radish Peroxidase (Sigma-Aldrich, St. Louis, USA, 1:5000 in BSA/PBS-T) and ECL Detection Reagent (GE Healthcare Ltd, Little Chalfont, Buckinghamshire, UK), followed by detection on a Luminescent Image Analyzer (LAS-3000, Fuji Photo Film Co., Ltd, Tokyo, Japan). Rats infected with 10, 25 or 50 ML with OD_n _values roughly equal to the average OD_n _in the *T.spiralis *ES ELISA were selected for testing in the western blot.

### Statistical analysis

The correlation between measured OD_n _values and ^10^log transformed values of recovered larvae per gram (LPG) were determined using Microsoft Excel statistical section. From the 2D normal distribution for OD_n _and LPG, low estimate (0.05 percentile) and high estimate (0.95 percentile) of the conditional distribution of LPG given the OD_n _value was determined.

For analysis of the dose effect on OD_n _per time point, the Generalized Linear Model (GLM) Fit of Mathematica 8.0.1.0 (Wolfram, Inc, Champaing IL, USA) was used. The combined effect of time and dose was calculated in the GLM using the formula OD_n _= *a*+*b*·dose·day. Probability levels less than 0.05 were considered significant.

## Results

### Clinical scores and body weight

**Figure 1:**
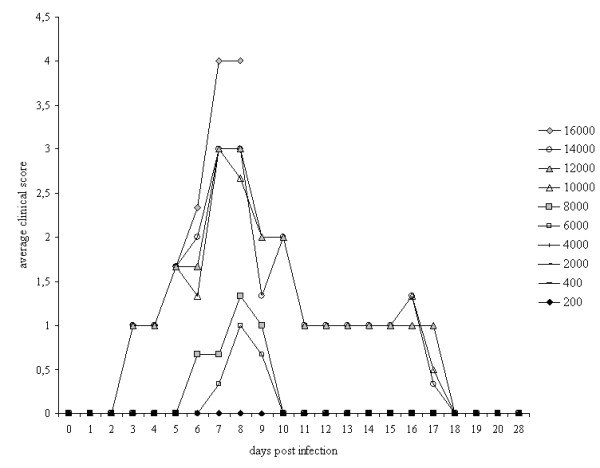
**Average clinical scores per dose group (*n *= 3)**. Clinical signs developed in animals that were infected with 6 000 ML or more, mainly during the intestinal phase of Trichinella. In animals that were infected with 10 000-16 000 ML, clinical symptoms set on earlier and lasted longer as compared to rats that received lower doses.

No clinical signs were observed in animals that were infected with less than 6 000 ML. The rats that were infected with 6 000 or 8 000 ML showed mild clinical symptoms (maximum average clinical score 1-1.25 on day 8 pi) from day 5 to day 10 pi. In animals that were infected with 10 000-14 000 ML, clinical symptoms started earlier (day 2 pi), lasted longer (day 18 pi) and the maximum average clinical score reached level 3 in these animals (Figure 1). The animals in the highest dose group (16 000 ML) were severely affected at day 8 pi (clinical score level 4, body weight loss 21 ± 2.6%, Figure 1) and it was decided to euthanize these animals at day 8 pi.

The average bodyweight of rats infected with 200-2000 ML showed no decrease after infection with *T. spiralis *ML. The animals in the higher infection dose groups lost bodyweight in a dose dependent manner [11]. Briefly, between day 5 and day 8 pi, the average bodyweight of the rats infected with 4 000 ML decreased with 7.2% ± 5.5%. Rats infected with 6 000-14 000 ML lost 15.8% ± 4.3% bodyweight. From day 10 onwards, the rats of the 4 000-14 000 ML infection groups regained bodyweight (129 g ± 24 g from 8 dpi to 42 dpi) at the same rate as the animals in the 200-2 000 ML groups (123 g ± 4 g).

### Pathology

The rats that received 16 000 ML were euthanized at day 8 pi and were necropsied immediately after euthanasia. All three animals showed hypermotility of the pale intestine. The duodenum up to the ascending colon was distended with clear, slightly viscous fluid containing minute flakes. Microscopically, shed villus tips were visible in these contents. The stomach contained only bile. Peyers patches and mesenteric lymph nodes were markedly enlarged. The spleen was small and the thymus very small. No body fat was present.

**Figure 2:**
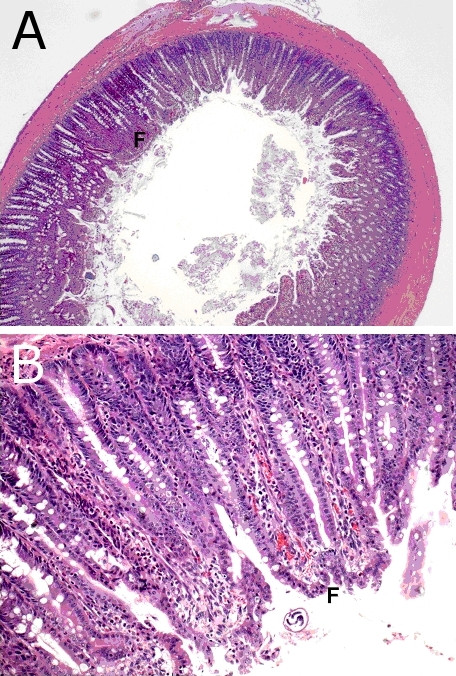
**A. Histopathology of duodenum at 8 dpi (16 000 ML, H&E)**. **A**. Villi are fused (F) and contain small hemorrhages as seen at magnification 20×. **B**. detail from A at magnification 100×.

**Figure 3:**
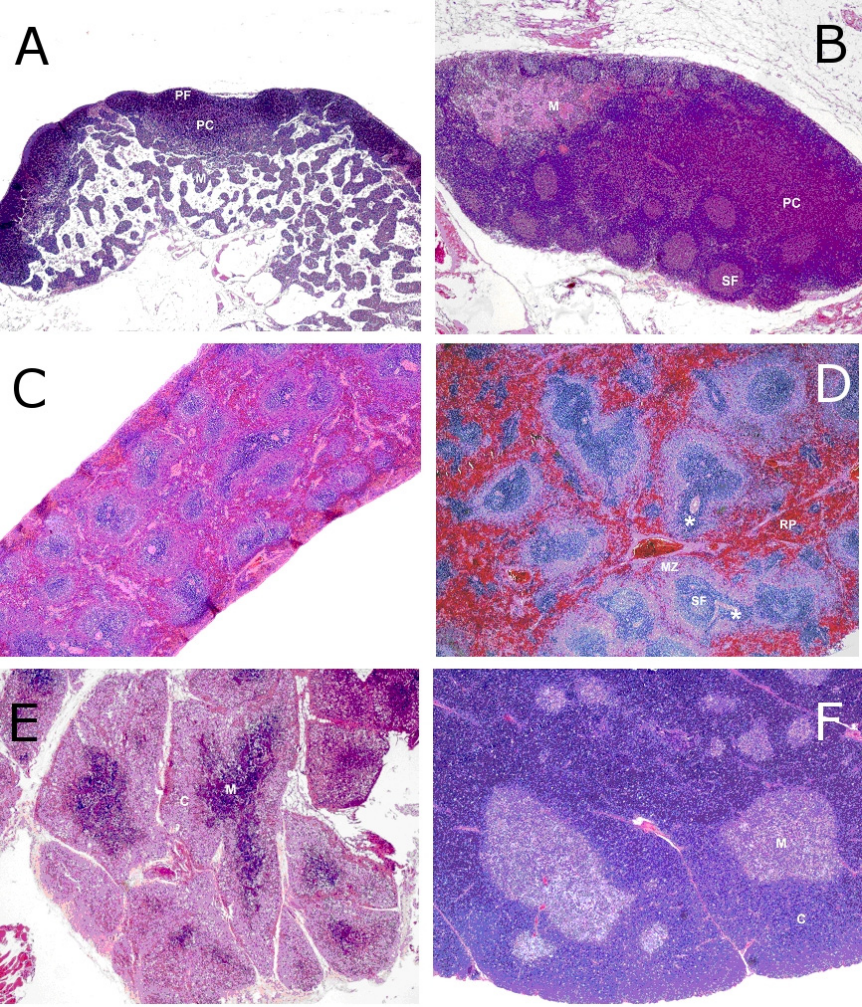
**Histopathology of rat lymphoid organs on day 8 pi (16 000 ML, left panel) and day 42 pi (14 000 ML, right panel), H&E, magnification 40×**. **A**. Mandibular lymph node 8 dpi, note small size, inactive (primary) follicles (PF) and paracortex (PC). **B**. Mandibular lymph node 42 dpi, activated appearance with multiple stimulated secundary follicles (SF), paracortex (PC) and medullary chords (M) **C**. Spleen 8 dpi, small and inactive appearance compared to **D**. Spleen 42 dpi, activated appearance: prominent secondary follicles (SF) and marginal zone (MZ) **E**. Thymus 8 dpi, smaller than F. with disappearance of cortex producing a reverse corticomedullary contrast compared to **F**. Thymus 42 dpi, prominent size and appearance with clear contrast between cortex (C) and medulla (M).

Histopathologically, intestinal lesions were most severe in the proximal small intestine and caecum. *Trichinella *females were most numerous in the proximal small intestine but present up to the descending colon. The mucosa of the duodenum and jejunum was covered with a thick layer of PAS-positive mucus containing extruded epithelial cells. Erosion was minimal due to obvious efficient fusion of the remaining short and distorted villi (Figure 2). Immaturity of the superficial epithelium was generally observed. Inflammatory infiltration in the *lamina propria *consisted mainly of eosinophilic leucocytes, many of which were degranulated. In the caecum, the submucosa was infiltrated and oedematous. Plasmacytosis of the medullary cords was responsible for the enlargement of the mesenteric lymph nodes. Other lymph nodes showed lymphoid atrophy (Figure 3a). Splenic peri-arteriolar lymphocyte sheath (PALS) and marginal zones were atrophic (Figure 3c). Follicle centres in the spleen and lymph nodes showed slight necrosis. In the thymus cortex, there was complete loss of lymphocytes (Figure 3e). No abnormalities were found in the liver, heart and lung. In the muscles, notably in the diaphragm, acute degeneration of myocytes was seen, together with some newborn larvae.

**Figure 4:**
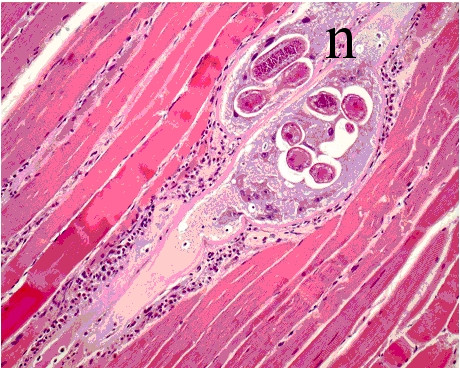
**Histopathology of muscle tissue at 42 dpi (14 000 ML, H&E, magnification 200×)**. Swollen transformed muscle fibers contain live muscle larvae (Nurse cells, n) surrounded by inflammatory cells (mainly macrophages).

At terminal sacrifice of the other animals on day 42 pi, no abnormalities in the heart, liver and duodenum were found in any of the infection groups. The cellularity in the lamina propria of the duodenum was prominent but no difference between the 200 and the 14 000 ML dose groups was observed. Mandibular lymph nodes showed an activated appearance as seen by abundant secondary follicles, paracortex and medullary cords (Figure 3b). The spleen was often congested with prominent marginal zones (Figure 3d). The thymus was well developed and showed no signs of regression (Figure 3f). Only a single muscle sample was taken per treatment group. The most prominent changes were seen in the highest dose groups. Individual myofibers were swollen and degenerated and contained pale amorphous material harboring larvae (nurse cells). Muscle fibers in the vicinity were often degenerated. Inflammatory cells (predominantly macrophages) were abundantly surrounding affected fibers, but typically not directly in or on the nurse cells (the larvae or capsules) (Figure 4).

No difference was found in lymphoid organs from the intermediate dose groups. The numbers of muscle larvae increased with infection dose as shown after counting, but these data were of limited quantitative value, as only 1 muscle sample per treatment group was taken, and significant variation per muscle sample is to be expected.

### Recovery of muscle larvae

Administration of 10 *T. spiralis *ML resulted in active infections in all animals from which on average 6.96 ± 4.42 larvae per gram (LPG) could be recovered. Increasing infection doses resulted in increasing numbers of recovered larvae, in a non-linear manner as was demonstrated in our laboratory [11]. Infection doses above 10 000 ML resulted in decreasing numbers of recovered muscle larvae at day 42 pi. No ML could be recovered on day 8 pi from muscle tissue of animals infected with 16 000 ML.

### Correlation between OD and LPG

**Figure 5:**
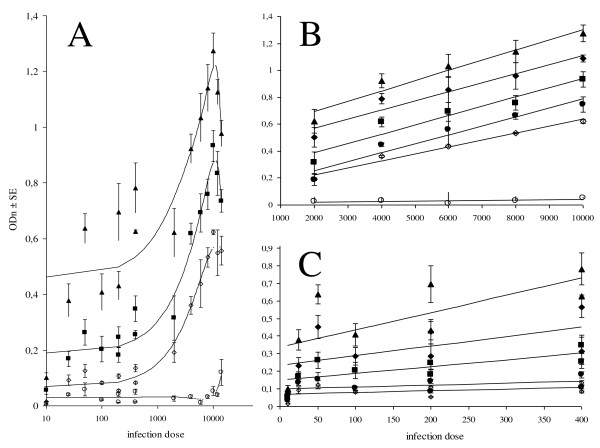
**Measured ODn values plotted against infection dose**. Immunological response on day 7 (open circle), day 14 (open diamond), day 21 (black circle), day 28 (black square), day 35 (black diamond) and day 42 (black triangle) pi. Infection doses 200 and 400 ML of both low and high dose experiments are displayed. **A**. Average OD_n _± SE values show a positive correlation with infection, both in time and infection dose, although less pronounced at low infection doses (10-400 ML). At the highest doses (12 000-14 000 ML), OD_n _values decline between day 14 and day 42 pi as compared to the 10 000 ML infection group. Intermediate time points are not shown for the sake of clarity. **B**. Average OD_n _values of infection groups 2 000-10 000 ML correlate significantly (*P *< 0.05) with infection dose on all time points **C**. Average OD_n _values of infection groups 10-400 ML correlate significantly (*P *< 0.05) with infection dose on all time points, except 7 dpi.

All experimentally infected rats seroconverted between 7 and 14 dpi with OD levels well above cut-off, except for the animals of the 10 ML infection group, which seroconverted between 14 and 21 dpi. In general, there is a positive correlation between OD_n _± SE with infection dose, although there is a remarkable difference between low (10-400 ML) and high (2 000-10 000 ML) infection dose ranges (Figure 5a). OD_n _values of rats that were infected with 12 000 and 14 000 ML declined as compared to the 10 000 ML infection group, reflecting the decline in LPG yield at 42 dpi in these infection groups (data not shown). There was a significant positive correlation between OD_n _values and infection dose in the range 2 000-10 000 ML at all time points (*P *< 0.05, Figure 5b). In the lower dose groups there was a significant positive correlation at all time points, except 7 dpi (*P *< 0.05, Figure 5c). The increase of OD_n _values over time was significant for all infection groups (*P *< 0.05).

**Figure 6:**
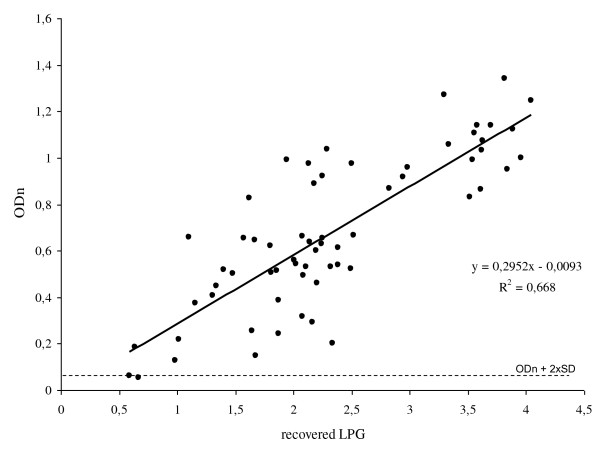
**Relation between antibody level and recovered LPG**. Individual OD_n _values were plotted against ^10^log transformed LPG, calculated from the number of larvae per rat that was isolated by artificial digestion. Data points represent individual animals of both the low- and high dose infection experiments. All but one point are above the cut-off level (OD_n _0.061).

OD_n _values of individual animals measured at 42 dpi plotted against ^10^log recovered LPG yielded a positive correlation (R^2 ^= 0.668, Figure 6). However, a wide range of LPG values corresponds to a specific OD_n _value (Table 1).

**Table 1:** Low estimate (0.05 percentile) and high estimate (0.95 percentile) of the conditional distribution of expected LPG given the OD value.

OD measured	Low estimate LPG	High estimate LPG
0	3	19
0, 1	5	31
0, 2	7	51
0, 3	12	84
0, 4	19	138
0, 5	31	228
0, 6	51	377
0, 7	84	623
0, 8	138	1029
0, 9	228	1700
1	376	2809
1, 1	621	4642
1, 2	1025	7672
1, 3	1694	12678
1, 4	2799	20953

### Western blot analysis

**Figure 7:**
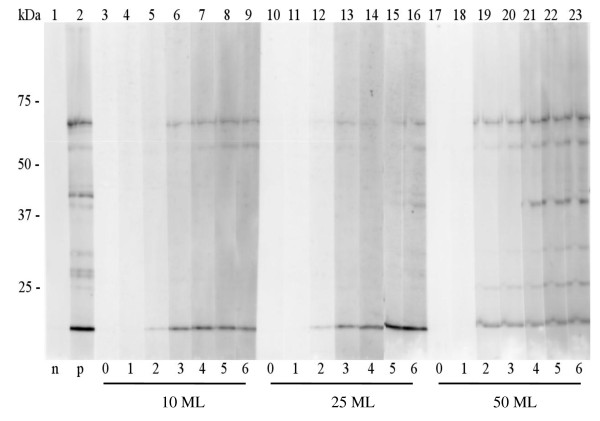
**Immune response of experimentally *T. spiralis *infected rats as determined in Western blot**. Lane 1: negative control (pooled pre-immune rat sera), lane 2: positive control (day 42 serum of a rat infected with 200 *T. spiralis *ML), lanes 3-9: experimental rat serum 0-6 weeks pi with 10 ML infection dose, lanes 10-16: experimental rat serum 0-6 weeks pi with 25 ML infection dose, lanes 17-23: experimental rat serum 0-6 weeks pi with 50 ML infection dose.

Infection with as little as 10 ML is sufficient to generate an IgG response on 14 dpi in the *T. spiralis *ES western blot. Although the signal was fairly weak with a serum dilution of 1:50, bands were clearly visible (Figure 7). Moreover, by using Western blot assay, seroconversion was detected at 14 dpi, which was one week earlier for this low infection level, as compared to ELISA. The seroconversion between day 14 and 21 pi as measured with ELISA was confirmed by an increase in signal with Western blot. With increasing infection dose, the number and intensity of recognized bands is enhanced.

## Discussion

The present study confirmed that *T. spiralis *is highly infective at very low doses in Wistar rats and can easily be detected by serology or pathogen detection using digestion. We describe the dynamics of *T. spiralis *infection in rats using doses ranging from very low (10 ML per rat) to very high (16 000 ML), with the aim of studying the usefulness of rats as sentinel animals in a wildlife monitoring program for *Trichinella*. Rats are sentinels for several human pathogens and have several advantages for epidemiological studies as mentioned by Psaroulaki et al. [14]. This is the first report describing the clinical, pathological, immunological and parasitological findings using a dose range of *T. spiralis *in experimental infection in rats this wide.

Infection with 10-2 000 ML caused no clinical symptoms in experimentally infected rats. Body weights of rats infected with 4 000 ML or more, declined in a dose-dependent manner until day 8-9 pi. This was probably caused by diarrhea and a lesser food intake during the intestinal phase of *T. spiralis *infection as observed before by Aulí and Fernández [15]. Although the probability of survival might have been biased by administering soft food, all animals, except the rats in the highest dose group (16 000 ML) were able to overcome illness, regained weight and survived until the end of the experiment.

This was corroborated by the differences in post mortem pathological findings between rats euthanized and examined at day 8 pi (16 000 ML infection group) and rats euthanized at day 42 (200-14 000 ML). During the intestinal phase of *T. spiralis *(day 8 pi, 16 000 ML), rats showed increased mucus production and intestinal hypermotility. This was in accordance with the model of Khan and Collins [16], which describes enteritis induced Th2 immune response resulting in intestinal muscle hyperactivity and increased mucus production by goblet cells.

Histological changes were most prominent in the duodenum at day 8 pi, while on day 42 the duodenum was not altered in any of the other infection groups. Also the thymus showed severe atrophy at day 8 pi, but showed no pathological alterations after 42 dpi in the other infection groups. Both features indicate successful regeneration of these organs after regression and inactivity indicative of severe biological stress. Findings in the spleen and (mandibular) lymph nodes seen on day 42 pi were considered indicative of systemic immune stimulation. The finding of wild caught *Rattus norvegicus *with very high LPG, ranging from 5 720 to 7 692 [5,7], indicates that also in nature wild rats are able to survive these high infection levels.

Infection with high doses of *T. spiralis *ML was characterized by a large variability in the numbers of muscle larvae that developed in individual rats. The maximum number of recovered muscle larvae was found in rats infected with 10 000 ML. Rats infected with 12 000 ML developed fewer muscle larvae and infection with 14 000 ML produced the fewest muscle larvae. This was probably due to severe competition for space (in the intestinal epithelium as well as in muscle fibers) and nutrients, in combination with expulsion of *T. spiralis *intestinal stages by the host. No ML could be recovered on day 8 pi from the muscle tissue of animals infected with 16 000 ML, although some ML could be identified in the diaphragm after histology. This might indicate that the intramuscular larvae might have been destroyed after digestion at this early developmental stage, since the protective nurse cell formation process starts at 8 dpi and is completed only on day 26 pi [17].

One of our goals was to determine whether ELISA was a suitable tool for detecting *T. spiralis *in rats. Therefore, it would be necessary to determine (1) the correlation between OD and infection dose in time and (2) the correlation between measured OD and exact numbers of *T. spiralis *ML in the rat. This is important to be able to evaluate ELISA results of future serological surveys in wild rats.

There was a clear antibody response in all infection groups. Seroconversion in rats infected with 25 ML or more, took place between days 7 and 14 pi, but already at day 7 pi, OD_n _levels of rats infected with 14 000-16 000 ML were above cut-off. Rats infected with 10 ML showed a gradual linear increase in OD above the cut-off level from 14 to 42 dpi.

We found a positive correlation between OD_n _values and infection dose, although less pronounced in the 10-400 ML infection groups. Some studies, also performed in rats, focused mainly on immune response with a limited number of infective doses. Salinas-Tobon et al. [18] performed experiments in rats with infection doses of 700, 2 000, 4 000 and 8 000 ML. Specific antibodies did not increase proportionally with the different ML doses tested, but the seroconversion period that occurred between 10 and 19 dpi, varied according to the infection dose.

We found a positive correlation between the number of recovered ML and serum antibody levels, although the predictive value of measured OD to estimate infection levels is limited, reflecting host variation in the immune response against a *Trichinella *infection.

The kinetics of anti-*T. spiralis *newborn larvae (NBL) immunity and its dose effects were studied in vivo by Wang [19]. In that study, rats were infected with 500, 2 000, 5 000 and 6 000 ML and immune response was measured as a reduction in the numbers of recoverable NBL after intravenous challenge with 10 000-100 000 NBL on day 16 pi. One of the results of that study was that infection with 2 000 ML *per os *induced the strongest immunity and that high dose immunization might induce a suppressive effect on host immunity. Our results demonstrate the exhausting effect on rats for doses above 8 000 ML and the more moderate clinical effects for doses of 6 000 and 8 000 ML. The lower immune effects with 5 000 and 6 000 ML observed by Wang [19] could reflect this strong impact on rat health rather than a real suppressive effect on the immune system. Serum antibodies were not measured in that study.

In our study, infection dose and OD correlated linearly at all time points after infection with 2 000-10 000 *T. spiralis *ML. Infection with lower doses resulted in a positive correlation with OD, but only after 14 dpi or more, and at a lower, though increasing level with time. Infection with higher doses (12 000 and 14 000 ML) resulted in lower numbers of recovered larvae as compared to the 10 000 ML infection group (70% and 60% respectively), which explains the observed comparable decline in OD_n _between 28 and 42 dpi. However, at day 7 pi the OD_n _values in the 12 000-16 000 ML infection groups are considerably higher than those of the 10 000 ML group.

Rats that were infected with 10, 25 or 50 ML showed a serological response in the *T. spiralis *western blot 2 weeks post infection. Other rodents like mice have been experimentally infected with low doses of 50, 10, and 5 ML of *Trichinella spiralis *per animal [20,21]. Seroconversion was observed in these experiments at 30 dpi with an ES-ELISA and specific antibodies increased until 80 dpi. Measured splenic T-lymphocyte activity increased from day 10 to day 15 pi, even with 10 ML as an infective dose [21]. This implies that for epidemiological studies, low infection levels can be detected by serology, both in rats and mice and we confirmed that low infection doses of *T. spiralis *larvae induce the production of specific antibodies at detectable levels in rats. These low doses reflect the infection level that we can find in the sylvatic cycle. Hurnikova and Dublinskyl [22] underlined that *Trichinella *(*britovi, pseudospiralis *and *spiralis*) infection in wild foxes is usually below 20 LPG and far less with wild boar. In The Netherlands, *Trichinella britovi *infection in wild foxes is even lower with LPG ranging from 0.04 to 0.71 [23]. For confirmation of rat sera from animals with low *Trichinella *infection levels or higher infection levels early in the time course of infection that exhibit OD values around the cut-off, we showed that western blots are a suitable instrument.

The results of our study confirmed previously conducted studies that were performed with very limited infection dose ranges in other host species like cattle [24], sheep [25,26], goats [27,28], horses [29-31], wild boars, pigs and foxes [32-34]. In these studies, the animals were experimentally infected with *T. spiralis *or other *Trichinella *species. As in our study, in most of these experiments, the time point of seroconversion and specific antibody titer were also dose dependent.

In summary, we show that rats, even infected with a low dose of 10 *Trichinella *ML, develop an immune response, which can be detected by use of serological assays and this immunological response is dose dependent up to an infection level of 10 000 ML. This indicates that the *Trichinella *ES-ELISA and Western blot are useful instruments for the detection of the presence of *T. spiralis *in sentinel populations like wild rats, which easily cross sylvatic-domestic borders. We also show that antibody levels can not be used to calculate exact LPG values in rats due to high variation in infection rates.

## Ethical Issues

Experimental infections in rats were conducted according to the Dutch laws applicable. The Central Animal Laboratory of the National Institute for Public Health and the Environment, the Netherlands, possesses a license under the Dutch 'Animal Experiments Act'. In accordance with Section 14 of this act, an officer has been appointed to supervise the welfare of laboratory animals.

## Competing interests

The authors declare that they have no competing interests.

## Authors' contributions

FFJF generated and analyzed parasitological and serological data, and wrote the first draft of the manuscript. MF and AG contributed to the generation of parasitological data. KT carried out the statistical analysis of the data. MAK and PWW carried out the pathological analysis-, and data interpretation. IV and PB contributed to the design and analysis of the studies including generation of parasitological data. JWBG conceived and designed the experiments, coordinated the study and contributed to the manuscript first draft. All authors read, approved and contributed to the final manuscript.
